# Thioridazine induces apoptosis by targeting the PI3K/Akt/mTOR pathway in cervical and endometrial cancer cells

**DOI:** 10.1007/s10495-012-0717-2

**Published:** 2012-03-30

**Authors:** Sokbom Kang, Seung Myung Dong, Boh-Ram Kim, Mi Sun Park, Barry Trink, Hyun-Jung Byun, Seung Bae Rho

**Affiliations:** 1 Research Institute, National Cancer Center, 323, Ilsan-ro, Ilsandong-gu, Goyang-si, Gyeonggi-do 410-769 Republic of Korea; 2 Division of Gynecologic Cancer Research, Research Institute and Hospital, National Cancer Center, 323, Ilsan-ro, Ilsandong-gu, Goyang-si, Gyeonggi-do 410-769 Republic of Korea; 3 Division of Head and Neck Cancer Research, Department of Otolaryngology and Head & Neck Surgery, The Johns Hopkins University School of Medicine, Baltimore, MD 21231 USA

**Keywords:** Thioridazine, Apoptosis, Anti-cancer activity, mTOR signaling, Cervical tumorigenesis

## Abstract

Recently, thioridazine (10-[2-(1-methyl-2-piperidyl) ethyl]-2-methylthiophenothiazine), a well-known anti-psychotic agent was found to have anti-cancer activity in cancer cells. However, the molecular mechanism of the agent in cellular signal pathways has not been well defined. Thioridazine significantly increased early- and late-stage apoptotic fraction in cervical and endometrial cancer cells, suggesting that suppression of cell growth by thioridazine was due to the induction of apoptosis. Cell cycle analysis indicated thioridazine induced the down-regulation of cyclin D1, cyclin A and CDK4, and the induction of p21 and p27, a cyclin-dependent kinase inhibitor. Additionally, we compared the influence of thioridazine with cisplatin used as a control, and similar patterns between the two drugs were observed in cervical and endometrial cancer cell lines. Furthermore, as expected, thioridazine successfully inhibited phosphorylation of Akt, phosphorylation of 4E-BP1 and phosphorylation of p70S6K, which is one of the best characterized targets of the mTOR complex cascade. These results suggest that thioridazine effectively suppresses tumor growth activity by targeting the PI3K/Akt/mTOR/p70S6K signaling pathway.

**Electronic supplementary material:**

The online version of this article (doi:10.1007/s10495-012-0717-2) contains supplementary material, which is available to authorized users.

## Introduction

Thioridazine (10-[2-(1-methyl-2-piperidyl) ethyl]-2-methylthiophenothiazine), a phenothiazine derivative, is a potent anti-psychotic and anti-anxiety agent belonging to the phenothiazine drug family. It is widely used to treat psychotic disorders such as psychosis and schizophrenia. Thioridazine may interact with other drugs which can cause drowsiness, such as alcohol, anti-depressants, muscle relaxants, anti-histamines, pain relievers and anxiety medicines. In case of advanced cancer, the drug has been used to treat cancer-related sweating [1, 2] and depression [3]. However, there have been several previous reports showing that the drug may cause serious side effects and a reversal of drug resistance, as well as, neuroleptic malignant syndrome [4–8]. Although several mechanisms, including inhibition of P-glycoprotein [9], DNA damage [10], or anti-oxidant activity [11], have been proposed to explain the anti-cancer effects of the drug, the true mechanism of thioridazine’s anti-cancer effects have been unclear.

Recently, a group of researchers observed that the anti-proliferative effect of chlorpromazine, another phenothiazine derivative, can be blocked by wortmannin, a selective PI3K inhibitor [12]. Thioridazine use may lead to the development of symptoms that resemble Parkinson’s disease, but these symptoms are not directly caused by Parkinson’s. In a study, we demonstrated the similiarities of the gene expression profile between thioridazine and known PI3K/Akt pathway inhibitors using the gene-expression-based query [13], while also showing that thioridazine could exhibit PI3K/Akt pathway inhibition in ovarian cancer cells.

Phosphatidylinositol 3-kinase (PI3K)/Akt signal transduction plays an important role in cell growth via inhibition of apoptosis in various types of human cancers [14–17]. Activation of Akt also promotes tumor metastasis and invasion, antagonizes cell-cycle arrest, angiogenesis, and phosphorylates mTOR (mammalian target of rapamycin) protein kinase. The mTOR pathway is mediated by a wide variety of cellular signal communications which include hormones, such as insulin and growth factors, nutrients, such as amino acids and glucose, and cellular stress conditions. The phosphorylation of Akt is modulated by phosphatidylinositol-3,4-bisphosphate and phosphatidylinositol-3,4,5-triphosphate, which are generated by (PI3K) [18]. PI3K is an activator of Akt, which consists of catalytic subunits (110 kDa) and regulatory subunits (85 kDa) [19]. A principal pathway that signals via mTOR is the PI3K/Akt signaling pathway, which is critically involved in the regulation of cell proliferation and survival. mTOR may also indirectly influence the phosphorylation condition of 4E-BP1 by modulating the activity of PP2A (Protein Phosphatase-2A). The second step effector, which is down-stream of mTOR, is the p70S6K serine/threonine kinase. After processing a cell proliferative up-stream signal mediated by the PI3K/Akt pathway, mTOR phosphorylates and activates p70S6K. mTOR plays a key role in the regulation of cell cycle progression, which includes protein synthesis, tumor growth, and angiogenesis [20–22].

In the present study, we estimated the anti-proliferative effect of thioridazine in human cervical and endometrial cancer cells, and determined the underlying molecular mechanisms. In addition, here we showed that thioridazine is a potent suppressor of cellular signaling pathways of PI3K-Akt-mTOR.

## Materials and methods

### Cell culture, antibodies, and chemicals

Human cervical (HeLa, C33A and Caski) and endometrial (HEC-1-A and KLE) cancer cell lines were maintained in DMEM (Life Technologies, Gaithersburg, MD) and RPMI1640 added with either 10 % heat-inactivated fetal bovine serum (FBS), penicillin (100 U/ml), or streptomycin (100 μg/ml), at 37 °C in a humidified atmosphere containing 5 % CO_2_. The following antibodies were used in this study: anti-Akt, anti-phospho-specific Akt, anti-PI3K, anti-phospho-specific PI3K, anti-cyclin A, anti-cyclin B1, anti-cyclin D1, anti-CDK1, anti-CDK2, anti-CDK4 (Santa Cruz Biotechnology, Santa Cruz, CA), anti-caspase-3, anti-Bcl-2, anti-Bcl-xL, anti-Bax, anti-mTOR, anti-phospho-mTOR, anti-p70S6K, and anti-phospho-p70S6K(Thr 421) (BDPhamingen, San Diego, CA), anti-p21 and anti-p27 (Oncogene, San Diego, CA), anti-4E-BP1 and anti-phospho-4E-BP1 (Cell Signaling) and anti-*β*-actin (Sigma). Rapamycin was purchased from Cell Signaling (Berverly, MA). Other chemicals and anticancer drugs were purchased from Sigma (St. Louis, MO).

### Cell viability assays

The viability of cell growth was determined using 3-(4,5-dimethylthiazol-2-yl)-2.5-diphenyl-^2^H-tetrazolium bromide (MTT) assays. Briefly, cell lines were grown in DMEM medium containing 10 % FBS. The cells were plated at a density of 3.4 × 10^3^ cells/well in 96-well plates. After 24 h, fresh medium containing 10 % FBS and 20 μl of MTT solution (Sigma, 5 mg/ml) was added to each well. Each well was then incubated for an additional 4 h at 37 °C. The amount of MTT-formazan generated was measured as the absorbance by using a microculture plate reader at 540 nm. Each individual measurement was repeated three times.

### Cell cycle analysis and apoptosis assays

Cancer cells were distributed onto six-well plates and then treated with thioridazine (15 μM) for 24 h, respectively. To evaluate apoptotic cells, the nuclei were fixed in methanol, stained with 2 μg/ml of 4,6′-diamidino-2-phenylindole (DAPI, Boehringer Mannheim, Mannhein, Germany) for 15 min at 37 °C, rinsed twice with PBS, and monitored under a fluorescence microscope (Zeiss; Switzerland). All experiments were carried out in triplicate. Subsequently, for the analysis of the DNA content using flow cytometry, each cell line was maintained at a density of 3.2 × 10^5^–4.1 × 10^5^ cells in 60-mm plates. After treatment with thioridazine, the cells were harvested, rinsed with ice-cold PBS, and fixed with ice-cold 70 % ethanol. The cells were centrifuged for 5 min at 1,000×*g* and resuspended in PBS containing 5 mM of EDTA and RNase A (1 mg/ml). After incubation for 1 h at 37 °C, the cells were treated for 15 min with fluorescein isothiocyanate (FITC)-labeled Annexin V and propidium iodide (PI), according to the supplier’s protocols (Boehringer Mannheim, Mannhein), and then were analyzed with a flow cytometer (FACScalibur, Becton–Dickinson, Franklin Lakes, NJ).

### Measurement of caspase-3 activity

For caspase-3 activity, cells (2.5 × 10^6^) were grown in either the absence or presence of thioridazine at 37 °C for 24 h. Caspase-3 activity was measured using an actyl-DEVD-7-amino-4-trifluoromethyl coumarin as the substrate, according to the manufacturer’s instructions (BDPharmingen, San Diego). In brief, the cells were placed with VP-16 (100 μg/ml) for 24 h, lysed in lysis buffer, and centrifuged for 25 min at 12,000×*g* at 4 °C. The activity was quantified in the supernatant fraction according to its proteolytic cleavage of the colorimetric substrate by use of a Spectramax 340 microplate reader (Molecular Devices, Sunnyvale) in fluorescence mode, with excitation at 405 nm and emission at 505 nm.

For assay of PARP cleavage, we carried out the procedures as described in the previous study [23, 24]. In brief, 50 μg of protein was placed with 60 μM biotinylated NAD in a 50 μl final volume of PARP reaction buffer (50 mM Tris–HCl, pH 8.0 and 25 mM MgCl_2_) at 37 °C for 1 h. The reaction was stopped by the addition of SDS loading dye buffer, and the products were separated using SDS-PAGE gel and autoradiography.

### Immunoblotting analysis

After treatment with thioridazine, cells were harvested by centrifugation. Cell extracts were prepared by washing cells with PBS, and cells were lysed in a buffer containing protease inhibitor. The protein yield was measured using the Bio-Rad protein assay kit. Equal amounts of protein were loaded, separated by SDS-PAGE gel, and then transferred to polyvinylidene difluoride membrane. After blocking, the membranes were placed at room temperature for 1 h with primary antibodies applied. The blots were washed thrice in wash buffer and incubated with the appropriate horseradish peroxidase-linked secondary antibodies. The immunoreactive bands were developed using the ECL detection system.

### Luciferase assays

Luciferase activity was carried out with a dual luciferase reporter assay kit (Promega, Madison, WI). Cancer cells were transfected by using the vector DNA containing p21-, p53- and Bcl-2-luciferase, in which the luciferase is expressed under each promoter control. The reporter plasmid, Bcl-2-Luc and p53-Luc were kindly provided by Dr. K. Park (Samsung Medical Center, Korea), and p21 promoter reporter construct by J. Park (Yonsei University, Korea). Data presented are representative of three replicate experiments. Briefly, cells at 80 % confluency were transiently transfected with each indicated reporter construct. After lysis with Reporter Lysis Buffer (Promega), lysates were cleared with centrifugation for 15 min at 14,000 rpm and cell extracts were incubated with the luciferase substrate reagent for 30 min at room temperature. Then, a 5 μl aliquot of each sample was transferred into the MicroLumat Plus LB96V luminometer. The ratio was normalized for the *Renilla* luciferase activity to correct for variations in transfection efficiency.

### PI3K assays

Enzyme assays were carried out as described previously by Fruman et al. [25]. In brief, cells were plated at a density of 1.8 × 10^6^ cells. After overnight incubation, cells were treated with either 15 μM thioridazine or wortmannin and LY294002 as a PI3K inhibitor, or left without treatment as the control for 24 h. Cells were lysed in 1 % NP-40 lysis buffer containing 20 mM Tris–HCl (pH 7.5), 100 mM NaCl, 1 mM EDTA, 1 mM MgCl_2_, 1 % NP-40, 1 mM phenylmethylsulfonyl fluoride, and 0.1 mM sodium orthovanadate. The lysates were centrifuged at 4 °C for 15 min at 20,000×*g* and the supernatants were used as the cell lysate. To immunoprecipitate PI3K, proteins were incubated for 1 h at 4 °C with anti-p85 antibody, followed by incubation with protein A-agarose beads for an additional 1 h at 4 °C. Immnunoprecipitates were incubated with kinase reaction buffer mix containing 200 μg/ml of phosphatidylinositol and 2 μCi of [-^32^P] ATP per assay mixture at 37 °C for 15 min. The reaction products were developed using autoradiography and the radioactive lipids were quantified by liquid scintillation counting.

### Statistical analyses

All data values were presented as either the mean ± SD (mean ± standard deviation) or means ± SEM (means ± standard error of means). Statistical comparisons were measured using Student’s *t*-test. Statistical analyses were carried out by using STATA software ver. 10.0 (StataCorp, College Station, TX). *P* values of <0.05 (*) were considered significant.

## Results

### Thioridazine regulates the cell proliferation and apoptosis

**Fig. 1:**
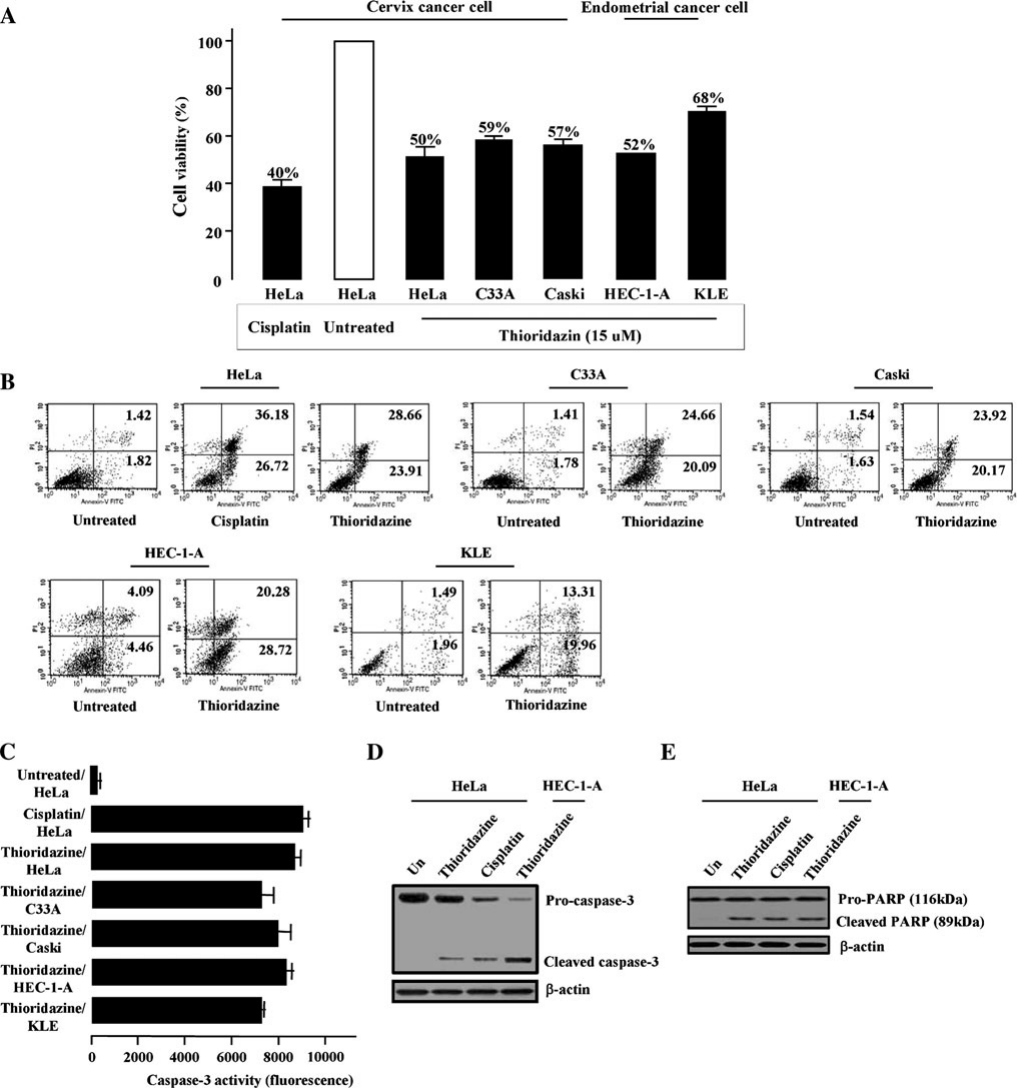
Treatment of thioridazine inhibits cellular proliferation and activates caspase-dependent pro-apoptotic activity. **a** Effect of treatment with thioridazine (15 μM) was analyzed by 3-(4,5-dimethylthiazol-2-yl)-2.5-diphenyl-^2^H-tetrazolium bromide (MTT) assays. Inhibition of cellular proliferation by cisplatin (20 μM) in HeLa cells was also illustrated for comparison. **b** Early- and late-stage apoptosis induced by thioridazine was analyzed by fluorescein isothiocyanate (FITC)-labeled Annexin V assay. **c** Caspase-3 enzymatic activity after treatment of thioridazine was determined using actyl-DEVD-7-amino-4-trifluoromethyl coumarin as the substrate. **d**, **e** Caspase-3 and PARP cleavages induced by cisplatin and thioridazine treatments. Soluble protein extracts were conducted by immunoblotting for cleaved caspase-3 and cleaved PARP. β-actin was used as an equal loading control

In order to examine the effect of thioridazine-induced inhibition of cellular proliferation in cervical and endometrial cancer cells, cells were treated with thioridazine (15 μM). As shown in Fig. 1a, cell viability of the cervical (HeLa, Caski and C33A) and endometrial (HEC-1-A and KLE) cancer cells were reduced by treatment of thioridazine. To confirm that the reduction in the cell numbers was reflective of cell death, flow cytometric detection was performed using labeled annexin V (Fig. 1b). Among all tested cell lines except KLE cells, cells treated with thioridazine demonstrated significantly increased early- and late-stage apoptotic fraction, which suggests that cell growth suppression by thioridazine was due to increased apoptosis. In addition, we compared the influence of thioridazine with cisplatin and similar apoptotic patterns between the two agents were observed in HeLa cells.

Subsequently, we assessed whether this effect of thioridazine is associated with an activation of caspase-3. In western blot analysis, as well as activity, thioridazine clearly induced activation of caspase-3. Especially in HeLa and HEC-1-A, the degree of caspase-3 activity was comparable to that of cisplatin (Fig. 1c). Additionally, cleavage of caspase-3 was also confirmed in response to thioridazine in HeLa and HEC-1-A, indicating that caspase-3 activation was mediated by cleavage of caspase-3 (Fig. 1d). We also compared the potency of thioridazine in cell proliferation inhibition with those of wortmannin and LY294002. As shown in Supplementary Fig. 1, proliferation of cells treated with wortmannin or LY294002 was inhibited to 53 and 52 % lower than the control in HeLa cells. In addition, thioridazine had more cell growth inhibitory effect than either of these two known PI3K inhibitors. Finally, the cleavage and activation of caspase-3 resulted in the characteristic proteolysis, e.g., cleavage of poly (ADP-ribose) polymerase (PARP) of HeLa and HEC-1-A after treatment of thioridazine (Fig. 1e). These results corresponded to our previous observation that thioridazine may inhibit cellular proliferation and induce apoptosis.

### Thioridazine-induced alteration of cell cycle modulators

**Fig. 2:**
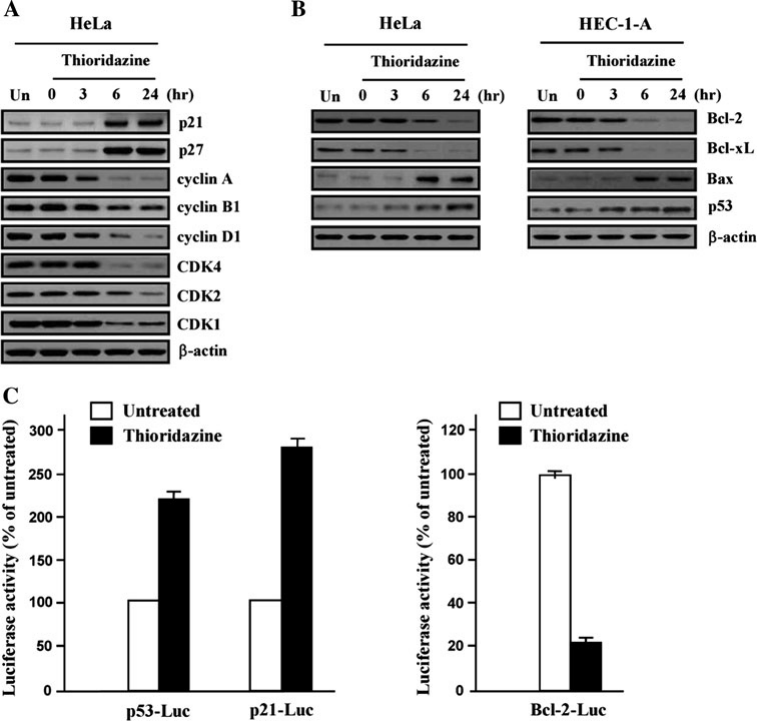
Effect of thioridazine on the expression of cell cycle regulatory protein and apoptosis-related genes. **a** Soluble protein extracts were subjected by western blot for the indicated proteins (p21, p27, cyclin A, B1, D1, CDK4, CDK2 and CDK1). **b** Expression of Bcl-2 family genes (Bcl-2, Bcl-xL, Bax and p53) were tested by using immunoblotting. β-actin was used as loading control. **c** After treatment with thioridazine, transfection with p53-, p21- and Bcl-2-luciferase, respectively. After collection by centrifugation, mixed with the reaction substrate and the effect on luciferase activity were measured by using a luminometer. All experiments were performed at least three times with similar results

Since we previously found that thioridazine may modulate the regulation of cell cycle progression by interfering with the PI3K/Akt pathway and induces G_1_ cell cycle arrest, we then investigated the influence of thioridazine treatment on changes to the cell cycle regulatory proteins using immunoblotting assays. As expected, we observed that thioridazine dramatically inhibited the expression of cyclin D1 and CDK4. In addition, p27 protein expression was enhanced after treatment of thioridazine, suggesting CDK inhibitor p27 has a major role in G_1_ cell cycle arrest induced by thioridazine. We also observed reduced expression of cyclin A and increased level of p21, which was consistent with a G_1_ cell cycle arrest. The expression of cyclin A and CDK2, which regulates transition from S or M phase, was decreased with the treatment of thioridazine. CDK1 and cyclin B1 regulate progressions from G_2_ to M phase, and were also inhibited (Fig. 2a).

Since p21 is the transcriptional target of p53, the expression of p53 was explored (Fig 2b). Expression level of p53 was increased after treatment of thioridazine along with Bax. In contrast, expression of anti-apoptotic protein Bcl-2 and Bcl-xL was decreased. Furthermore, by using luciferase reporter assay, we confirmed that thioridazine increased transcription of p53 (Fig. 2c). In the Supplementary Fig. 2, p21 and p27 more enhanced the expression levels of wortmannin-treated HeLa cells when compared with that of thioridazine-treated cells as expected. Also, treatment of thioridazine or wortmannin significantly increased the levels of Bax and p53 expression in a time-dependent manner. Thus, it can be speculated that thioridazine induces G_1_ cell cycle arrest by increasing transcription of p53 and its transcriptional target, p21.

### Suppression of PI3K/Akt/mTOR/p70S6K phosphorylation by thioridazine in cervical and endometrial cancer cells

**Fig. 3:**
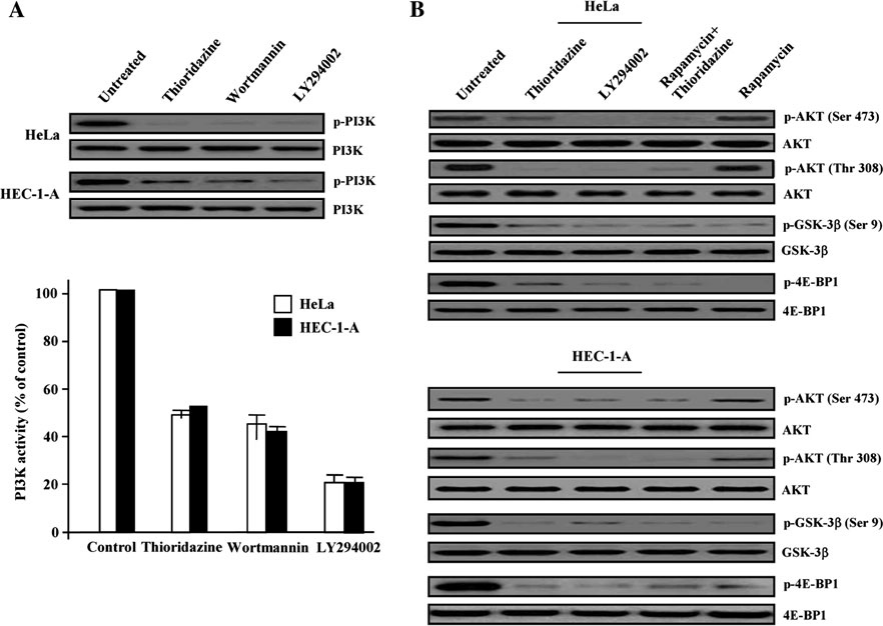
Thioridazine promotes pro-apoptotic signaling through inhibition of p-Akt activation. **a** Effect of thioridazine and other PI3K inhibitors on PI3K activity was assayed by immunoblotting for total PI3K and phosphorylated PI3K. The reaction products were visualized using autoradiography and liquid scintillation counting. **b** HeLa and HEC-1-A cells were treated with each chemical drugs and analyzed for the indicated proteins by immunoblotting for total Akt/phosphorylated Akt (p-Ser 473 and p-Thr 308), GSK-3β/phosphorylated-GSK-3β (p-Ser 9) and 4E-BP1/phosphorylated-4E-BP1. Results shown are representative of three independent experiments

We tested whether thioridazine could induce inhibition of PI3K activity in HeLa and HEC-1-A cells. After treatment with EGF (50 ng/ml, 6 h), cell lysates were immunoprecipitated using anti-p85 antibody with or without thioridazine treatment. After treatment of thioridazine, cells showed significantly inhibited phosphorylation of PI3K. This inhibitory activity was comparable to that of two well-known PI3K inhibitor, wortmannin and LY294002 (Fig. 3a). The inhibition of PI3K in the two cells resulted in inhibition of Akt, which is one of the major downstream targets of PI3K. As expected, thioridazine also successfully inhibited phosphorylation of Akt (Ser 473) and phosphorylation of 4E-BP1 (Fig. 3b), one of the best characterized targets of the mTOR complex. Also, treatment with thioridazine decreased the level of phosphorylated Akt (Thr 308) as well as the level of phosphorylated GSK-3β (Ser 9) within 3–6 h, whereas wortmannin and LY294002 significantly inhibited the level of phosphorylated protein within 6 h (Supplementary Fig. 3). When thioridazine was co-treated with rapamycin, inhibitor of mTOR, we observed that they exerted additive effects in the deactivation of p-4E-BP1. These results suggest that confirm our former hypothesis that thioridazine is a potent inhibitor of PI3K activity.

**Fig. 4:**
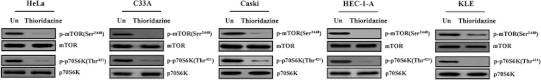
Western blot analysis of down-stream components in the Akt signaling pathways with thioridazine-treated cervix cancer and endometrial cancer cells, respectively. After treatment of thioridazine, total cells lysates were prepared and investigated for phospho-mTOR(Ser^2448^) and phospho-p70S6K(Thr^421^) protein levels by immnunoblot analysis. Unphosphorylation protein was used as an equal loading control (indicated as mTOR and p70S6K)

Finally, after treating the five cell lines (three cervical and two endometrial) with EGF (50 ng/ml, 6 h), we examined the effect of thioridazine on the down-stream signaling components in the PI3K/Akt pathway. Thioridazine inhibited the phosphorylation of mTOR on Ser 2448 residue and p70S6K on Thr 421 residue, a downstream target of mTOR (Fig. 4). Taken together, our results support the idea that thioridazine targets the Akt/mTOR/p70S6K signaling pathway, which leads to the inhibition of tumor growth and metastasis.

## Discussion

The current study has shown that thioridazine inhibits cellular proliferation through the induction of G_1_ cell cycle arrest and cellular apoptosis and that the anti-proliferative effect of thioridazine on cervical and endometrial cells may result from the inhibition of PI3K/Akt/mTOR/p70S6K signaling pathways by thioridazine, which corresponds with our previous study [26].

The PI3K/Akt/mTOR/p70S6K signaling pathways play a pivotal role in the physiological functions of human malignant tumors. PI3K modulates signaling pathways implicated in cell growth, apoptosis, or both. Regulatory factors of this pathway are frequently deregulated in an extensive number of tumor types, making it an attractive target for tumor therapy [27]. Akt activity is modulated by PI3K, which recruits Akt to the cell membrane, permitting its activation by PDK1 [28]. Akt activation induces cell cycle progression, survival, migration and metabolism through phosphorylation of various physiological factors. Activation of PI3K and Akt are reported to occur in ovarian, thyroid, breast and pancreatic tumor [29].

Based on these findings, the PI3K/Akt pathway is believed to be a promising therapeutic target for the treatment of cancer. Moreover, a body of evidence indicates that inhibition of PI3K/Akt pathway may inhibit cell growth, and increase the cytotoxic effect of conventional chemotherapeutic agents in many solid tumors [17, 30–34]. In addition, mTOR, a major down-stream target of PI3K/Akt pathway, is well-known as an essential regulator of tumor growth and cell proliferation, protein synthesis, and the modulation of signals in various signaling pathways [27, 35].

Thus, our data suggest that thioridazine alone or with conventional cytotoxic agents may be worth to be tested in further studies as an effective therapeutic option. The anticancer effect of thioridazine had been demonstrated in vivo by using a mouse model system [36]. In addition, although there has been no well-designed human trial, there was a case report of successful treatment with high-dose thioridazine in a cervical cancer patient [37]. Moreover, thioridazine has already been used in cancer patients for managing depression and psychosis [38]. However, because the drug is not free from side effects, such as cardiac toxicity, movement disorder, and central nervous system effect, the toxicity of high-dose thioridazine should be carefully evaluated. In addition, biologically achievable dose and specificity of the drug should be determined further. Since there have been several case reports addressing the clinical efficacy of phenothiazine derivatives in cancer patients [37, 39], the clinical use of thioridazine as a targeting agent of PI3K/Akt/mTOR signaling pathway may not be unrealistic when these concerns are resolved by further research.

In summary, our data show that thioridazine can inhibit the PI3K/Akt/mTOR/p70S6K signaling pathway and exert cytotoxic effect on cervical and endometrial cancer cells by inducing cell cycle arrest and apoptosis. The usefulness of this agent in cancer treatment should be explored in future research.

## Electronic supplementary material

Below is the link to the electronic supplementary material.

Supplementary material 1 (DOC 1744 kb)

## Abbreviations

Thioridazine: 10-[2-(1-methyl-2-piperidyl) ethyl]-2-methylthiophenothiazine
mTOR: The mammalian target of rapamycin
CDK: Cyclin dependent kinase
MTT: 3-(4,5-dimethylthiazol-2-yl)-2.5-diphenyl-^2^H-tetrazolium bromide
FITC: Fluorescein isothiocyanate
PI: Propidium iodide
