# Supplementary material for: Thioridazine induces apoptosis by targeting the PI3K/Akt/mTOR pathway in cervical and endometrial cancer cells
Source: Apoptosis. 2012 Mar 30;17(9):989–97. doi: 10.1007/s10495-012-0717-2 (PMC3413814; doi:10.1007/s10495-012-0717-2)
Supplement: Supplementary file 1 — Supplementary material 1 (DOC 1744 kb) [file 10495_2012_717_MOESM1_ESM.doc]

**Materials and methods**

*Antibody*

The following antibodies were used in this study: anti-GSK-3β, anti-phospho-specific GSK-3β (Ser 9), anti-Akt and anti-phospho-specific Akt (Thr 307) (Santa Cruz Biotechnology, Santa Cruz, CA) was purchased from Cell Signaling (Berverly, MA). Other chemicals and anticancer drugs were purchased from Sigma (St. Louis, MO).

**Figure legends**

**Fig. S1** Treatment of cisplatin, thioridazine, wortmannin, or LY294002 inhibits cellular proliferation and activates caspase-dependent pro-apoptotic activity. **a** Effect of treatment with thioridazine was analyzed by 3-(4,5-dimethylthiazol-2-yl)-2.5-diphenyl-^2^H-tetrazolium bromide (MTT) assays. Inhibition of cellular proliferation by cisplatin, wortmannin or LY294002 in HeLa and HEC-1-A cells were also illustrated for comparison. **b** Early- and late-stage apoptosis induced by cisplatin, wortmannin or LY294002 as well as thioridazine were analyzed by fluorescein isothiocyanate (FITC)-labeled Annexin V assay. **c** Caspase-3 enzymatic activity after treatment of cisplatin, thioridazine, wortmannin or LY294002 were determined using actyl-DEVD-7-amino-4-trifluoromethyl coumarin as the substrate.

**Fig. S2** Effect of thioridazine and wortmannin on the expression of cell cycle regulatory protein and apoptosis-related genes. Cells were treated to compare with a time-course of shorter times (0, 3, 6, 24 h) using thioridazine or wortmannin in HeLa cancer cells, respectively. **a** Soluble protein extracts were subjected by western blot for the indicated proteins (p21, p27, cyclin A, B1, D1, CDK4, CDK2 and CDK1). **b** Expression of Bcl-2 family genes (Bcl-2, Bcl-xL, Bax and p53) were tested by using immunoblotting. β-actin was used as loading control.

**Fig. S3** Western blot analysis of down-stream components in the PI3K signaling pathways. Cells were treated to compare with a time-course of shorter times (0, 3, 6, 24 h) using thioridazine, wortmannin or LY294002 in HeLa cancer cells, respectively. After treatment with thioridazine, wortmannin or LY294002, cells were harvested, dissolved in lysis buffer. The cell lysates were subjected to immnunoblotting with the indicated antibodies. Unphosphorylation protein was used as an equal loading control (indicated as PI3K, Akt and GSK-3β).


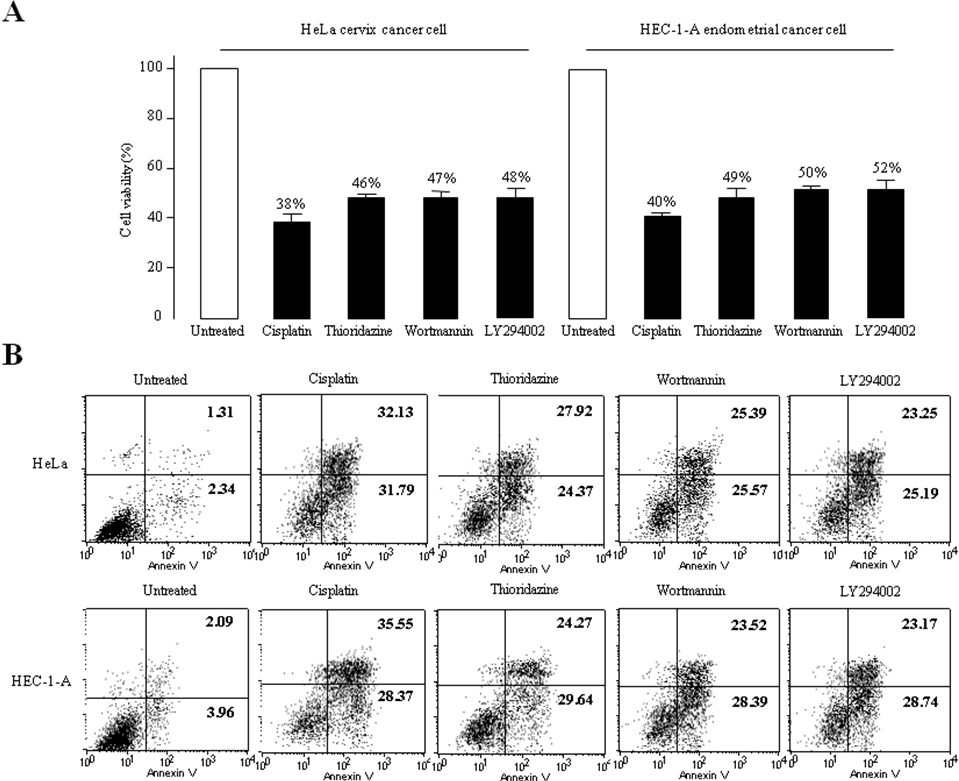


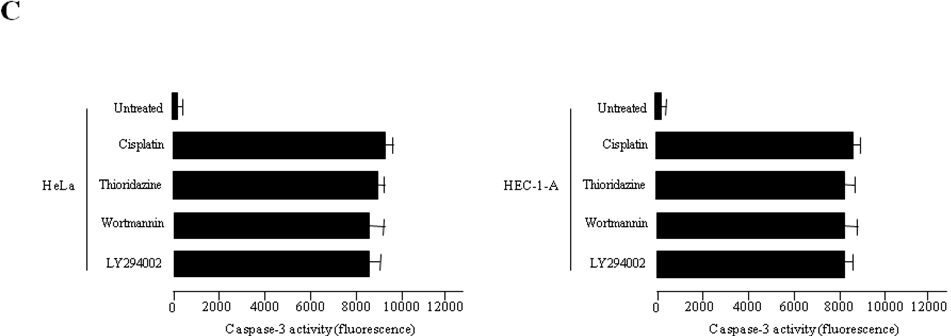


Supplementary Fig. 1


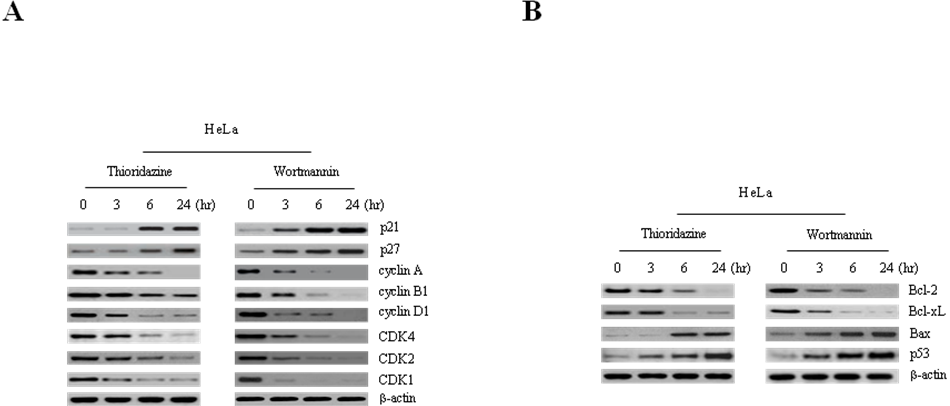


Supplementary Fig. 2


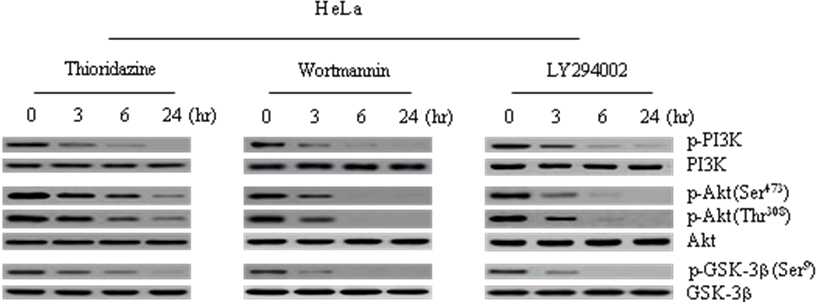


Supplementary Fig. 3
